# Historical reconstruction of Latin American psychosomatic medicine: the exchange between psychoanalysts from Argentina and Brazil, 1942-1959

**DOI:** 10.1590/S0104-59702024000100046en

**Published:** 2024-10-11

**Authors:** Carla Ribeiro Guedes, Adriana Kaulino

**Affiliations:** i Professor, Departamento de Saúde e Sociedade/Instituto de Saúde Coletiva/Universidade Federal Fluminense. Niterói – RJ – Brazil carlaguedes@id.uff.br; ii Professor, Facultad de Psicología/Universidad Diego Portales. Santiago – Chile adriana.kaulino@udp.cl

**Keywords:** Psychosomatic medicine, Institutionalization, Psychoanalysis, History

## Abstract

This article reconstructs the origins of psychosomatic medicine in Latin America through the history of the institutionalization of psychoanalysis in Argentina and Brazil. The study focuses on the exchange between Argentine and Brazilian psychoanalysts from 1942 to 1959. The analyses indicate similarities and differences between the two projects of dissemination and theoretical production of psychosomatic medicine in both countries. The conclusions indicate that the Argentine group of psychoanalysts had the intention of strengthening psychoanalysis while, in Brazil, the main disseminator of psychosomatics, Danilo Perestrello, supported a profound transformation of traditional medicine.

In 1902, the first disciples of Sigmund Freud began meetings in the famous “Wednesday psychological afternoons.” It was a group formed mostly by doctors and intellectuals dissatisfied with the problems related to human subjectivity in the then Victorian Vienna. In 1908, this group was renamed Psychoanalytic Society, and, in 1910, the International Psychoanalytical Association (IPA) was founded (Rose, 1992).

Over the years, with the growing social recognition of Freud and psychoanalysis, several psychoanalytic societies were founded around the world. Starting in 1920, a complex process of institutionalization of psychoanalysis began in the Berlin Psychoanalytic Polyclinic (transformed into an institute), under the direction of Max Eitingon. Due to the great demand for psychoanalytic treatment, there was a significant change in the professional training of new analysts, which acquired an agility that made it more impersonal and standardized. It can be said that the rebellious character of world psychoanalysis of the first two decades was modified in the middle of the second by more conservative and reactionary tendencies (Vetö Honorato, 2013).

In turn, the persecutions caused by Nazism and the outbreak of the Second World War produced a geographical, doctrinal, and institutional dispersion, shifting its center of production from Central European to Anglo-Saxon scientific culture. Most Jewish psychoanalysts emigrated to the United States, where they founded psychoanalytic institutions strongly subordinated to the IPA (Plotkin, 2009; Vetö Honorato, 2013).

IPA, under the presidency of Ernest Jones (also president of the British Psychoanalytic Society), together with the American Psychoanalytic Association came to strongly control psychoanalysis with an orientation that considered it a neutral, pure, and apolitical science with an exclusively therapeutic approach. The psychoanalytic diaspora leaves behind the cultural, social, humanistic, and even subversive character of psychoanalysis, becoming bureaucratized and closed in a rigid organizational hierarchy (Roazen, 1973; Vetö Honorato, 2013).

In the Latin American region, the Argentine Psychoanalytic Association [Asociación Psicoanalítica Argentina, APA] was formally recognized by IPA in 1942. Following the postwar transformations in which psychoanalysis lost its humanistic character in favor of a strongly clinical-therapeutic aspect, APA was in tune with the theoretical orientations of the United States and England during its first years. This trend was articulated with the rapid growth of psychosomatic medicine, based on the American model (Plotkin, 2003).

Psychosomatic disorders began to be addressed by psychoanalysis in the first decade of the twentieth century with Sandor Ferenczi and George Groddeck. In the 1930s, at the Chicago Psychoanalytic Institute, the proposal for psychosomatic medicine emerged. The Hungarian doctor and psychoanalyst Franz Alexander and the American doctor Helen Flanders Dunbar are its main representatives. Together, they founded the first journal specifically dedicated to the subject in 1939, the *Journal of Psychosomatic Medicine*. Both, in their way, contributed to studies of the origins of pathologies based on psychoanalytic formulations (Birman, 1980).

Dunbar’s works stated that each illness corresponded to a psychological profile, built from basic childhood experiences (Dunbar, 1943). While, for Alexander, illnesses were linked to unconscious conflicts, that is, to a specific conflict. Alexander addressed seven pathologies: bronchial asthma, gastric ulcer, rheumatoid arthritis, ulcerative rectocolitis, dermatosis, thyrotoxicosis, and essential hypertension (Alexander, French, 1948).

The tradition of psychosomatic medicine exerted a profound influence on Argentine psychoanalysts in the process of establishing the APA, which can be evidenced in the publications of Franz Alexander in the inaugural issue of the *Revista de Psicoanálisis* [Journal of Psychoanalysis] (Alexander, 1943) and the first book produced by the APA library (Alexander, French, 1948). In its beginnings, the theoretical approach associated with the professionalization model of psychoanalysis, in the United States, had a significant impact on the members of the new association (Plotkin, 2003; Dagfal, 2018).

This chain of influences continues with the impact of the development of Argentine institutional psychoanalysis on psychoanalytic groups in other Latin American countries such as Brazil. Many first-generation Brazilian psychoanalysts, affiliated with IPA, did their training in Buenos Aires, including some doctors from Rio de Janeiro, such as Danilo Perestrello, considered the precursor of psychosomatic medicine in Brazil (Guedes, Rangel, Camargo Jr., 2020).

Despite the importance of the chain of psychosomatic medicine in the institutional origins of psychoanalysis in Argentina and Brazil, there are still no studies that address the configuration of psychosomatics in the two countries from a historical and institutional perspective. Until now, references to psychosomatics are usually linked to the history of psychoanalysis, locating its roots in Anglo-Saxon or European centers. Therefore, while in recent decades historical studies of various theoretical-methodological natures regarding psychoanalysis have proliferated, the development of narratives that reconstruct the historical particularities of psychosomatics in Latin America is scarce.

Considering the importance of psychosomatic medicine and the scarcity of studies on its history in the region, the article reconstructs its origins through the history of the institutionalization of psychoanalysis in Argentina and Brazil. The research focused on the institutionalization of psychoanalysis corresponds to a methodological strategy whose objective is to identify the appropriation of psychosomatics in both countries. In theoretical terms, the article shares the arguments according to which psychoanalysis would be a transnational system of ideas and beliefs (Porras, Báguena, 2020; Plotkin, 2017) which is integrated into “psi knowledge” (Rose, 1996). Its origins are varied, multiple, and sociocultural located in spaces different from the formation of official psychoanalytic societies. Consequently, this work focuses on reconstructing only one of the relevant processes in the historical configuration of psychosomatics: its relationship with the institutionalization and legitimation mechanisms of psychoanalysis in the sociopolitical contexts of Argentina and Brazil.

The study covers the period that begins with the foundation of APA in 1942 and ends with the creation of the Brazilian Society of Psychoanalysis of Rio de Janeiro, in 1959.

The primary sources of information were magazines, newspapers, conferences, articles, and books published by psychoanalysts that addressed topics related to psychosomatic medicine in Argentina and Brazil during this period. The discursive productions related to psychosomatic medicine present in newspapers, books, and other publications of APA *Revista de Psicoanálisis* were analyzed, as well as conferences, texts, and books by the Brazilian psychoanalyst trained by the Argentine association, Danilo Perestrello, from 1942 to 1959.

Also, secondary sources were used to reconstruct the institutionalization of psychoanalysis in both countries. For this purpose, manuscripts and books on the history of the institutionalization of psychoanalysis in Latin America and the United States were reviewed (Roazen, 1973; Bálan, 1991; Oliveira, 2000, 2017; Russo, 2002; Plotkin, 2003; Facchinetti, Ponte, 2003; Dagfal, 2009, 2018; Vetö Honorato, 2013; Ruperthuz, 2015; Facchinetti, Castro, 2015; Briolotti, 2016). Finally, secondary sources were analyzed that allowed us to understand aspects of the relevant social and political contexts during the period addressed by the study (Plotkin, 2009; Fiorucci, 2004).

## The institutionalization of psychoanalysis in Argentina

Psychosomatic medicine was one of the topics that interested the young pediatrician Arnaldo Rascovsky who in the 1930s promoted, in his office on Sundays, an informal meeting for friends and family, together with his wife Matilde Wenceblatt to read and discuss Freud’s works. The couple Enrique Pichón-Rivière and Arminda Aberastury were among the participants (Plotkin, 2003).

Discussions to form a psychoanalytic association affiliated with IPA began in the following decade, in 1940, initially with the project of transforming psychoanalysis into a medical specialty. This proposal failed, but two years later, APA was created in a movement led by the Spanish Ángel Garma. Although all the founders had a doctor’s degree, in the beginning, non-medical members were also accepted such as wives (Balán, 1991; Plotkin, 2003).

The original group was made up of doctors with fresh ideas about psychoanalysis but very different from each other. Rascovsky was a young Jewish pediatrician respected by the Jewish middle class of Buenos Aires and worked at the Children’s Hospital (Hospital de Niños). Pichon-Rivière was born in Switzerland, emigrated to Argentina with his parents when he was a child, graduated in medicine, and maintained relations with the artistic circles of the Buenos Aires avant-garde. When he began attending study meetings on psychoanalysis, he was already a young psychiatrist who worked at the Hospice of Mercedes (Hospicio de las Mercedes) and was well-established in the public psychiatric care system. Ángel Garma emigrated to Buenos Aires in 1938, the same year in which he finished his psychoanalytic training, in Berlin, with Theodor Reik as his trained analyst.

Garma was a member of the International Association and arrived in Buenos Aires with important psychoanalytic credentials. Céles Cárcamo was an Argentine doctor, who met Garma while he was undergoing training at the Paris Psychoanalytic Association. In 1939, Cárcamo returned definitively to Buenos Aires with a degree provided by IPA (Balán, 1991; Plotkin, 2003).

In 1942, APA received provisional recognition from IPA, while its official affiliation only occurred in 1949 during the first post-war psychoanalytic congress held in Zurich (Balán, 1991; Plotkin, 2003).

At first, APA attempted to establish closer ties with the local medical circuit. However, Argentine psychoanalysts ended up staying away from the medical establishment, with rare participation in conferences organized outside the local psychoanalytic circuit (Plotkin, 2003). The participation in conferences in Rio de Janeiro and São Paulo is among these exceptions as well as in a medical congress held in Rio de Janeiro (Marchon, 2012; Dagfal, 2018).

In July 1943, eight months after its founding, APA began publishing the *Revista de Psicoanálisis*. This journal was an important instrument for the dissemination, legitimation, and validation of APA. The small group of psychoanalysts in the new association, which resembled a “secret society,” needed to obtain recognition in specialized circles (Dagfal, 2018). Until the late 1950s, the work of its psychoanalysts rarely appeared in publications outside of APA journal (Plotkin, 2003). Once again, Rio de Janeiro escaped the rule, when *Revista Evolução Psiquiátrica* published an article by Arnaldo Rascovsky and Ludovico Rosenthal (1947) on the Latin American psychoanalytic movement that was also published in APA *Revista de Psicoanálisis*.

According to Plotkin (2003), this institutional isolation can be understood by relating it to the organizational and training conditions of APA, as well as the professional status of psychoanalysts who were affiliated with a private institution without ties to the traditional medical structure. Pichon-Rivière was the only one of the group who had established connections with the Buenos Aires psychiatric establishment. On the other hand, the psychoanalyst members of the association were linked to an international organization and were part of a network of relationships globally.

The institutional isolation of APA and the success of its development cannot be understood outside the political context of the time. The 1930s and 1940s had a series of political transformations in Argentina. The coup d’état of 1930 put an end to a period of institutional stability and was followed by international events such as the Spanish Civil War, the advent of Nazism in Germany, the radicalization of fascism in Italy, and the Second World War. At the national level, the military coup of 1943 produced a climate of polarization having two political and cultural groups: one progressive liberal and the other nationalist-Catholic. Perón’s government in 1945 expanded this political radicalization. His opponents formed a heterogeneous coalition, from socialists and communists to liberals and even some conservatives. Politics came to have a strong emotional nuance, in which people were divided between being Peronist or anti-Peronist (Plotkin, 2003).

The Peronist regime considered avant-garde art, existentialism, and other currents of thought, considered modern, to be anti-national, anti-popular, and, therefore, anti-Peronist. In this historical context, psychoanalysis was understood as part of the system of cultural resistance to Peronism. The early members of APA identified with the liberal tradition that was threatened. Garma had participated in the Spanish Republican Center [Centro Republicano Español], Pichón-Rivière had a socialist background and Marie Langer had belonged to the communist party in Vienna. Although psychoanalysts were not explicitly persecuted, they always had police at APA meetings (Plotkin, 2003).

The loss of prestige and legitimacy of academic institutions was another consequence of the Argentine political environment. The military government of 1930 eliminated university autonomy by decree and with the 1943 coup, figures from the nationalist and Catholic right were placed in charge of the universities who, among other measures, dismissed many professors for purely political reasons. The arrival of the Perón government, in 1945, further deepened the intervention of the State in the universities. This scenario contributed to the development of an intense intellectual life outside the university environment. APA gained legitimacy by managing to maintain its scientific and cultural autonomy (Plotkin, 2003).

Even in this adverse scenario, in the first years, the members of APA sought to expand their radius of influence. The dissemination strategies included the free distribution of the *Revista de Psicoanálisis* among doctors and other liberal professionals, as well as aggressive dissemination abroad (Dagfal, 2018). For our study, it is interesting to note that the first issue of the journal, published in 1943, opens with an article by the doctor and psychoanalyst Franz Alexander, one of the pioneers of psychosomatic medicine at the Chicago Psychoanalytic Institute.

## Psychosomatic medicine in the Argentine Psychoanalytic Association

The strategic position of Alexander’s text, titled “Psychological aspects of medicine,” in the inaugural issue of the *Revista de Psicoanálisis*, and *Factores psicógenos en el asma bronquial* [Psychogenic factors in bronchial asthma], the first book published by APA Library of Psychoanalysis, also by this author but this time in collaboration with Thomas French, both from 1943, reflect the impact caused by psychosomatic medicine on Argentine psychoanalysts.

One of the particularities of the first stages of psychoanalysis reception in the United States was its integration into medical and university discourse, as well as its great professionalization limited to medical practice. In this way, psychoanalysis was converted into a medical discipline absorbed by the psychiatric establishment, in which it was quite popular for young psychiatrists to undergo official psychoanalytic training (Vetö Honorato, 2013).

Although APA remained disconnected until the end of the 1950s from the Argentine medical environment, the professionalization of psychoanalysis was projected, in its beginnings, by its founders as something strictly medical and related to psychosomatic medicine. Even in 1948, APA began to require a medical degree for those who wanted to practice psychoanalysis (Balán, 1991; Plotkin, 2003).

Thus, APA came increasingly closer to psychoanalysis of the American model. The proposal defended by Alexander was the advent of a new medicine with a psychoanalytic basis that would rescue the vision of the human being as a unitary whole and not as a disease. It is curious to observe how the mind-body integration perspective of psychosomatics once again introduces humanistic rhetoric into the psychoanalytic discourse that wants to penetrate the world of medicine.

In the opening article of the *Revista de Psicoanálisis*, Alexander (1943, p.63) states: “The patient, considered again as a human being, with his/her worries, fears, hopes and despairs, as an indivisible whole and not only as a carrier of organs – of a diseased liver or stomach – is becoming a legitimate object of medical interest.”

In this passage, we can see the emergence of a “scientific psychological medicine” that dedicated more and more attention to the causal role of emotional factors in illness.

From 1942 to the late 1950s, the initial group of APA was interested in psychosomatic research, whether in theoretical or clinical approaches. The first two decades were dominated by the dissemination of events and publications on the topic. The main milestones include the holding of the course on psychosomatic problems, taught at the Children’s Hospital in 1943 (Dagfal, 2018), the congress on “headaches” in 1953, and the symposium on “obesity and eating disorders” in 1955, in which most of its members presented papers (Aizenberg, 1982). Also notable in this period is the publication of the work *Patología psicosomática* [Psychosomatic pathology] by Arnaldo Rascovsky (1948), the first book on the area compiled by a member of the institution.

Argentine psychoanalysts started from a declaration of principles constituted by the basic idea that every illness was psychosomatic (Aizenberg, 1982). In this regard, the members of APA differed from the perspective proposed by Alexander that some diseases were psychosomatic, and others were not: “This justified didactic attempt by Alexander does not exclude, in our opinion, in any way the concept of psychogenic participation in all pathological conditions, as well as in the understanding of the individual considered normal” (Rascovsky, 1948, p.11).

The great growth of the proposal of psychosomatic medicine within Argentine psychoanalysis in these first decades is explained, because this perspective largely offered an almost unlimited field of clinical interventions in which traditional medicine had not been successful (Plotkin, 2003).

As an illustration, Arnaldo Rascovsky and Luis Rascovsky (1945) carried out research from a psychoanalytic approach, with 116 cases of childhood epilepsy over five years in the outpatient clinic of the Neuropsychiatry and endocrinology service of the Children’s Hospital of Buenos Aires. The approach consisted of periodic interviews with caregivers of children who had epileptic seizures, to collect environmental and psychological data on family dynamics. The authors concluded that the most constant situation that precipitated epileptic seizures was generated by a deeply neurotic family organization, and by the fact that children shared a bed with one or both parents (or substitutes). They observed that simply removing the child from the bed led to the disappearance of epileptic seizures or a reduction in their number and intensity. From the point of view of the psychodynamic hypothesis, they stated that the elaboration of the experienced stimuli depends on the ability of the “self” to cope with the stimulation received:

The access occurs in situations in which the ego is overwhelmed by instinctive stimulating quantities that it cannot channel through the oral, anal, or genital organizational systems and whose magnitude is sufficiently intense to demand and provoke a type of somatic discharge even before oral organization; which is equivalent to saying that the epileptic attack constitutes a reaction pattern before the integration of the postnatal self (Rascovsky, Rascovsky, 1945, p.627).

Continuing with the trend of addressing clinical studies and/or theoretical debates, among the books and articles published by the association’s psychoanalysts and their guests, some studies cover a wide range of diseases related to different medical specialties: asthma (Racker, 1948), coryza (Ferrari, 1944), dermatological diseases (Allendy, 1946), epilepsy (Rascovsky, Rascovsky, 1945; Pichon-Rivière, 1944; Cesio, 1952), headache and migraine (Cárcamo, 1945; Garma, 1958), gynecological and sexual manifestations (Cárcamo, Langer, 1944; Langer, 1951), endocrine problems (Rascovsky, 1948), cough (Fenichel, 1944) and gastric ulcers (Garma, 1945, 1954).

It is interesting to note that in these works no affiliation to any specific school of thought in psychosomatic medicine can be identified. For example, in the 1948 article, “Basic concepts in psychosomatic medicine,” in which Pichon-Rivière (1983) relies on notions from Alexander, Kubel, and Fenichel, each with different or even antagonistic perspectives on the topic (Aizenberg, 1982).

The plurality of authors and theoretical approaches used in the articles points to the fact that the influence of the current American psychosomatic medicine, especially in the first decade of APA, did not correspond decisively to an affiliation with the Chicago Psychoanalytic School. From the first issue of APA *Revista de Psicoanálisis*, the prominence of Franz Alexander was shared with Melanie Klein, the Austrian psychoanalyst, living in England and who led a powerful current of followers in the British Psychoanalytic Society. During the 1950s and 1960s, Klein expanded her domain to Latin American countries such as Argentina, Brazil, and Chile (Dagfal, 2018; Oliveira, 2017; Vetö Honorato, 2013). However, although a Kleinian clinical hegemony occurred in Buenos Aires, it can be said that Argentine psychosomatic medicine remained characterized by its theoretical eclecticism (Aizenberg, 1982).

In 1955, the coup d’état that defeated Perón produced a political and sociocultural renewal in Argentina, with the democratization and modernization of the university and the expansion of the activities of psychoanalysts in public health services. In this new political context, at the end of the 1950s, members of APA began to work in previously unthinkable institutional settings, such as the Medicine School of the University of Buenos Aires [Facultad de Medicina de la Universidad de Buenos Aires] and public hospitals (Dagfal, 2009).

In line with the expansion of the activities of psychoanalysts in hospital services, medical-psychological interconsultation was developed addressing medical specialties such as gynecology and obstetrics, endocrinology, surgical psychoprophylaxis, dermatology, cardiology, and pediatrics (Aizenberg, 1982), and new proposals such as psychosomatic pediatrics (Briolotti, 2016).

The 1940s and 1950s are also characterized by the expansion of APA in Latin America, with the training of psychoanalysts from different countries and a close exchange between APA members and psychoanalysts from Rio de Janeiro (Russo, 2002).

## The exchange between psychoanalysts from Argentina and Brazil

Unlike what happened in Argentina, where psychoanalytic doctors were locked into a self-referential group within the psychoanalytic association, in Rio de Janeiro the big names that made up the psychiatric establishment were already propagating psychoanalysis in the public service, linked to pedagogical and hygienic projects (Russo, 2002).

Upon assuming power in 1930, Getúlio Vargas launched a program of social and cultural modernization, intellectually much more inclusive than the one from Perón in 1945. Vargas’s regime created a strong and centralized State, to work with intellectuals, regardless of their ideological tendency, in a broad program of political and cultural reforms. This program corresponded to the image constructed by many intellectuals, most of them from the families of the Brazilian elite, feeling like the mentors of uneducated and incapable people. Thus, the introduction of psychoanalysis in official educational institutions and hospitals was more accessible to Brazilian doctors and educators than to Argentine professionals (Fiorucci, 2004).

In this context of the 1940s, some young psychiatrists linked to the National Service for Mental Illnesses [Servicio Nacional de Enfermedades Mentales] were dissatisfied with the official orientation of that body and with the teaching of the Medicine School, which they considered outdated. The criticism fell on Henrique Roxo (1877-1969), a professor of psychiatry since 1911 and responsible for introducing Freudian theory into his university courses (Facchinetti, Ponte, 2003).

In 1944, this group of psychiatrists created the Juliano Moreira Study Center [Centro de Estudios Juliano Moreira, CEJM]. Some of its founders were Danilo Perestrello and Walderedo Ismael de Oliveira, who were later joined by Marialzira Perestrello and other psychiatrists. The CEJM aimed to bring together those interested in the study of Freud’s work and implement the training of psychoanalysts in Rio de Janeiro, based on the standards of IPA. In this sense, its members worked with two alternatives: bringing a trained psychoanalyst to Rio de Janeiro or undertaking analytical training abroad (Marchon, 2012).

Initially, the group sought to attract an APA educator who was interested in emigrating to Rio de Janeiro. Arnaldo Rascovsky and Ángel Garma were invited. Although the proposal did not obtain the expected result, both psychoanalysts came to Rio de Janeiro for a series of conferences for the CEJM. Rascovsky in July 1945 and Garma in January 1946. Rascovsky also met the group of psychoanalysts who were participating in the process of reconstitution of the psychoanalytic society of São Paulo (Marchon, 2012; Dagfal, 2018).

In his conference in Rio de Janeiro, Arnaldo Rascovsky (1947) referred to the creation of IPA in Europe, the United States, and Argentina. He showed great enthusiasm for the American training model linked to medical schools and the medical profession, pointing out the possibility of a similar path in Latin America with the development of psychosomatic medicine in medical sectors. Garma in his conference and showing harmony with his colleagues also addressed topics of psychosomatic medicine (Marchon, 2012).

The presence of Argentine analysts in the country strengthened the spirit to look for a foreign training analyst who wanted to come to Rio de Janeiro. Garma advised the group in his invitation to Georg Gerö. He also invited Marie Langer and the French psychoanalyst Daniel Lagache. However, none of the proposals succeeded (Marchon, 2012).

The first Inter-American Congress of Medicine, held in Rio de Janeiro in 1946, was a new opportunity for exchange between psychoanalysts from Brazil and Argentina. Regarding the preparations for this event, Marie Langer (1964, p.89-90) commented on the efforts of one of the founders of APA to spread psychoanalysis among Brazilians: “Rascovsky, a man very capable of propaganda and convincing, suggested that we all go to participate in the event”.

Several Argentine psychoanalysts participated in the congress: Ángel Garma, Céles Cárcamo, Marie Langer, Matilde Wencelblat, Eduardo Krapf, Enrique Pichon-Rivière, Arnaldo and Luis Rascovsky, Alberto Taglieferro, Arminda Aberastury, Flora Scolni, Teodoro Schlossberg, Horacio Garcia Vega, in addition to the patron of APA, Francisco “Paco” Muñoz (Marchon, 2012; Dagfal, 2018). At the congress, the Argentine group presented 17 works, contrasting with the three presented by the Brazilians Durval Marcondes, Virginia Bicudo, and Darcy de Mendonça Uchôa, all of them supervised by the educational analyst Adelheid Koch, from the São Paulo group, which had recently obtained the provisional recognition of IPA (Dagfal, 2018; Oliveira, 2017). This movement of the Argentines seems to demonstrate the political investment of the APA in the dissemination of psychoanalysis in Brazil, especially in Rio de Janeiro.^1^

At this same congress, Danilo Perestrello (1987) gave a conference on psychosomatic medicine entitled “Importance of the psychological factor in the etiopathogenesis of gastroduodenal ulcers.” In this presentation, he explained the genesis of somatic diseases from unconscious conflicts, using notions based on psychoanalytic theories of Franz Alexander and the Argentine Ángel Garma. In this way, we observed in the Brazilian context the creation of a theoretical framework that established the bases of organic diseases based on psychoanalytic theory, combining the contributions of the Chicago Psychoanalytic Institute with those of the psychoanalysts of APA. The success of the visit of Argentine analysts to the CEJM the previous year, with the dissemination of the notions of psychosomatic medicine, began to bear its first fruits.

The strengthening of relations between the groups in Rio de Janeiro and Argentina, together with the difficulty of bringing a foreign teaching analyst, caused some CEJM participants to seek transfer abroad. This was the case of Alcyon Baer Bahia, a doctor from the National Service for Mental Illnesses who had gone to Buenos Aires in 1945 to undergo training at APA. Since 1944, Mário Martins, a psychiatrist from Porto Alegre, and his wife Zaira Martins were already in training at APA.

Danilo Perestrello, Marialzira Perestrello, and Walderedo Ismael de Oliveira took advantage of the Rio de Janeiro congress to talk to didacts present at the event about the beginning of their analyses with APA (Facchinetti, Ponte, 2003). In 1946, the couple Marialzira and Danillo Perestrello embarked for Buenos Aires to begin their respective analyses, joined by Walderedo Ismael de Oliveira a few months later, in February 1947 (Marchon, 2012).

Training in a psychoanalytic association affiliated with IPA had very strict, hierarchical, and elitist rules, as it was very expensive in terms of time and money. In the 1940s and 1950s, the requirements imposed for obtaining the title of psychoanalyst were three hundred hours of didactic analysis, attendance, and approval of seminars for three or four years, treatment of two or three complete cases under the supervision of a teaching analyst, and presentation of a research monograph. After all this process, the candidate could be accepted as an associate member, and only after a few more years of practice and new theoretical work could finally be admitted as a full member of the association. The pinnacle of the psychoanalytic hierarchy consisted of being elected a didact member, that is, an analyst who was authorized to train other psychoanalytic candidates, without necessarily having to be in analysis (Plotkin, 2003).

The four young doctors from Rio de Janeiro had obtained a license funded by the National Service for Mental Illnesses, which allowed them to cover the high costs of analytical training dedicated exclusively to their qualifications (Facchinetti, Ponte, 2003). After the training, the didactic analyses and supervision began. Danilo Perestrello was analyzed by Cárcamo and supervised by Pichon-Rivière and Marie Langer; Marialzira Perestrello had Pichon-Rivière as analyst and was supervised by Garma and Cárcamo; Walderedo had Marie Langer as analyst and Garma and Arnaldo Rascovsky as supervisors; and Bahía was initially analyzed by Garma (the analysis was interrupted) and later by Cárcamo, with the supervision of Luis Rascovsky (Marchon, 2012).

The repetition of the same names as analysts and supervisors is striking. This situation also occurred at APA Institute, where the seminars were given by a select group participating Arnaldo Rascovsky, Enrique Pichon-Rivière, Angel Garma, Céles Cárcamo, Mary Langer, Arminda Aberastury, Luis Rascovsky, Alberto Talliaferro, Luiza Alvarez de Toledo, Teodoro Schlossberg, and E. Racker (since 1949) (Marchon, 2012).

Analysts had enormous power within each association. While they administered the didactic analyses, they were supervisors, teachers, and evaluators of the candidates. In a small association, this type of organization could generate confusing situations since a teaching analyst who knew the most intimate secrets of a candidate was the one who evaluated his/her academic performance (Plotkin, 2003).

This was the setting in which the analysts from Rio de Janeiro carried out their training at APA. In addition to the mandatory seminars focused on topics related to psychoanalytic theory and technique, the applicability of psychosomatic medicine was present in the Psychosomatic Service of the Children’s Hospital, where Bahia was an assistant (Marchon, 2012). However, Danilo Perestrello became primarily responsible for carrying the legacy of psychoanalytic contributions to medicine in Brazil (Guedes, Rangel, Camargo Jr., 2020).

While still in training in Buenos Aires, in 1948, Perestrello began to disseminate the notions of psychosomatic medicine with a presentation on emotions and colitis, in the strictly Latin American medical field at an event in Buenos Aires (see Perestello, 1958a).

## The institutionalization of psychoanalysis in Brazil

Danilo and Marialzira Perestrello returned to Brazil in 1949, Alcyon B. Bahía and Walderedo Ismael de Oliveira in 1950, when they created a group that became known in psychoanalytic circles as “the Argentines” (Marchon, 2012).

On the other hand, the training of psychoanalysts had only begun in Rio de Janeiro in 1948, first with the arrival of the psychoanalyst Mark Burke, a Polish Jew, member of the British Psychoanalytic Society, and later, Werner Kemper, who was a member of the Berlin Institute, even after it was “Aryanized” by the Nazi State. In 1936, after several acts of persecution against Jews who practiced psychoanalysis and against the institution of psychoanalysis, the Nazi government forced the German Psychoanalytic Society to disaffiliate from IPA and join the Göring Institute (German Institute for Psychological Research and Psychotherapy). The terms “psychoanalysis,” “psychoanalyst” and the entire psychoanalytic vocabulary were eliminated from the public scene. Although they were prohibited from practicing psychoanalysis, both in teaching and in the clinic, several psychoanalysts remained at the Göring Institute. Werner Kemper was one of them. After spending 12 years submitting to the demands of the Nazi regime, claiming that he would be protecting psychoanalysis, Kemper arrived in Rio de Janeiro with his wife, Kattrin Kemper, to create an official psychoanalytic society (Russo, 2002).

Two years after the arrival of the two trained analysts, the first dissidence occurred. Kemper’s group broke with the group linked to Burke, creating two training societies in a city where an official association did not yet exist. The accusations between the two groups were mutual and the reasons for the breakup were attributed to political issues related to Burke’s Jewish origin and Kemper’s conduct during the Nazi regime, as well as to the dispute between the German and English schools of psychoanalysis. In any case, what can be stated is that a dispute began between the two groups for the official recognition of IPA (Russo, 2002).

Kemper’s group sought support from the Brazilian Society of Psychoanalysis of São Paulo for the recognition of their study group. The Brazilian Society of Psychoanalysis was founded by Durval Marcondes, in 1927, in the capital of São Paulo state. However, the partnership was dissolved shortly thereafter. The process of its reconstitution began in 1937, with the arrival in São Paulo of the German psychoanalyst Adelheid Koch, linked to the Berlin Psychoanalytic Institute. Of Jewish origin, Koch suffered Nazi persecution and ended up accepting Ernest Jones’s proposal to emigrate to the city of São Paulo to organize the local psychoanalysis group and assume the role of teaching analyst. In the following year, the first educational analysis of Latin America began. The provisional recognition as a study group by IPA occurred in 1946. Its definitive recognition as a psychoanalytic society affiliated with IPA will occur in 1951 (Oliveira, 2000; Facchinetti, Ponte, 2003).

The support of the Koch group was the first step in the constitution of a Rio de Janeiro training society that obtained, in 1955, authorization to become the Psychoanalytic Society of Rio de Janeiro. People linked to Burke (who returned to England in 1953) joined the analysts trained in Argentina and in 1957 were recognized by IPA as a study group. Two years later, in 1959, this group was recognized as the Brazilian Society of Psychoanalysis of Rio de Janeiro. Marialzira and Danilo Perestrello belonged to this last group (Russo, 2002).

The Perestrello couple’s project included the occupation of the Brazilian Society of Psychoanalysis of Rio de Janeiro and the claim of a leadership position in the history of psychoanalysis in Rio de Janeiro. In a book written by Marialzira Perestrello (1987), she said that the psychoanalytic movement is divided between the “predecessors” and the “pioneers.” The first group would be formed by the disseminators of psychoanalysis in Rio de Janeiro and the second would be composed of those responsible for introducing psychoanalytic training in Brazil based on the guidelines of IPA. This differentiation was intended to demonstrate that the “pioneers” had been more important in defining the theory, practice, and study of psychoanalysis in Rio de Janeiro than their predecessors, that is, the psychoanalytic psychiatrists who, in the 1920s, were those responsible for the first translations of Freud’s work in the country. By constructing this historical narrative, the Perestrello and their group were in the position of pioneers committed to the dissemination of “true” psychoanalysis (Facchinetti, Castro, 2015).

It is interesting to observe how this narrative generates an “origin myth” (Danziger, 1979) for Brazilian psychoanalysis. The term myth indicates the weakness of a historical interpretation that claims to be true and legitimizes some situations of the present through the past. Origin myths are reiterated in traditional historical interpretations of psychoanalysis. From this historiographic perspective, psychoanalysis is attributed to a “legitimately recognized organizational life” (Ruperthuz, 2021, p.718). In traditional historiographies, the use of the concept of pioneer is frequent, particularly in local histories of psychoanalysis. The appeal to pioneers contributes to demarcating boundaries between prehistory and the beginning of the history of official psychoanalysis. The pioneers are distinguished both from their predecessors and the dissidents by their fidelity and theoretical contribution to supposedly true Freudian psychoanalysis. Consequently, these historical narratives often highlight the importance of pioneers in the founding of psychoanalytic societies officially recognized by IPA.

The history prepared by Marialzira Perestrello is a clear example of this historiographic strategy of legitimation of official psychoanalysis. However, in this case, the myth of origin has been revealed by various works that provide evidence that the differences between “pioneers” and “predecessors” are highly questionable (Facchinetti, Castro, 2015; Castro, 2017). Thus, the story that Marialzira Perestrello writes would be a strategy in the fight to establish the true heirs or representatives of a theoretical proposal in which the author attributes the prominence of psychoanalysis in Rio de Janeiro to her group (Russo, 1993).

## Psychosomatic medicine in Rio de Janeiro

While the different groups strove to obtain institutional recognition from IPA for the founding of the psychoanalytic society, Danilo Perestrello became the main disseminator of Brazilian psychosomatic medicine, assuming prominence and almost exclusivity, in the 1940s and 1950s, of the publications on the topic (Guedes, Rangel, Camargo Jr., 2020). This is different from the Argentine group since most APA psychoanalysts wrote about psychosomatics in its official dissemination platform, the *Revista de Psicoanálisis* (Dagfal, 2018).

In the case of Perestrello, his strategies to propose psychosomatic medicine visible were his conferences at events, national and international congresses in the medical area, as well as classes and oral communications in universities and hospitals, which were compiled in the book *Medicina psicossomática*, published in 1958, considered the inaugural work of this field in the Brazilian context (Guedes, Rangel, Camargo Jr., 2020).

Throughout these two decades, Perestrello dedicated to making psychoanalytic contributions to explain the causality of different somatic pathologies (Perestrello, 1958c), such as ulcers (Perestrello, D., 1987), headaches (Perestrello, 1958d, 1954), ailments dermatological diseases (Perestrello, 1958e) and digestive diseases (Perestrello, 1958f, 1958g).

In these works, the Argentine masters Garma, Rascovsky, Pichon-Rivière, and Cárcamo, as well as the Americans Alexander and Dunbar, were used as theoretical references, however, the influence of the French psychoanalyst Pierre Marty, who, together with Michel de M’Uzan, created the Paris School of Psychosomatics, is also noticeable in his work from the 1950s (Perestrello, 1958d). The French school breaks with the Chicago psychoanalytic school by conceiving the psychosomatic fact as disconnected from medical nosography and research into the personalities corresponding to psychosomatic diseases (Marty, M’Uzan, 1963).

Beyond clinical issues, Perestrello defends the project that psychoanalysis can contribute to the emergence of a “new medicine.” According to him, medicine and clinical practice had to be overcome through a gradual transformation of the organicist medical model towards psychosomatic medicine (Guedes, Rangel, Camargo Jr., 2020). In this new paradigm, the object of medicine would become the “patient as a person” and the patient’s psychic life would become valuable for understanding somatic illness (Perestrello, 1958b). In this way, the overcoming of organic medicine would be done through a humanist path through a psychoanalytically based medicine (Perestrello, 1958b).

Thus, Franz Alexander’s proposal to return to the humanistic perspective of psychoanalysis to dialogue with medicine is a central argument in Perestrello’s project.

In 1956, in his first course in psychosomatic medicine as a psychiatry chair at the Medical School of the Universidade do Brasil (current Universidade Federal do Rio de Janeiro), Perestrello presented the American model of inclusion of psychoanalysis in medical schools. The psychoanalyst argued that, to modify medical practice, it was necessary to invest in the teaching and institutionalization of psychosomatic medicine as the United States did: “At Columbia University, for example, the ‘Clinic for Psychosomatic Medicine and Psychoanalysis’ was created. for teaching and research purposes, under the direction of professors at the New York Psychoanalytic Institute; and today, most North American medical schools teach psychiatry in all years of the medical course” (Perestrello, 1958h, p.32; emphasis in the original).

Thus, Perestrello discusses the idea of creating the discipline of psychosomatics or medical psychology in medical courses to provide the student with basic psychological knowledge. However, for him, this measure would only be a transition, because, in the future, medicine would be psychosomatic and all medical courses would follow this orientation.

Perestrello was also dedicated to raising awareness among doctors so that the proposal of psychosomatic medicine could be applied in clinical medicine. The author expounded six principles of psychosomatic medicine that should guide clinical practice: (1) the doctor’s object of study is the sick man and not the disease; (2) there are no local diseases, every disease is general and affects the individual as a whole; (3) the isolated individual is an abstraction and can only be conceived in his environment; (4) emotional states can disturb the functioning of any organ and are as effective as physical stimuli in producing somatic modifications; (5) unconscious conflicts are mainly responsible for somatic symptoms; (6) emotional disorders can cause organic lesions (Perestrello, 1958b).

Throughout the years, the approach to medical practice acquired new contours in Perestrello’s career and was also related to a humanistic perspective of the doctor/patient relationship, especially from the influence of the book *The Doctor, his patient, and the illness*, by Michael Balint (1958) *.*

Although it is true that, mainly from the 1960s onwards, the theoretical and clinical references of Melaine Klein, together with the reading of the work of Wilfred Bion, guided the practices of Brazilian societies linked to IPA (Oliveira, 2017), Perestrello’s writings on psychosomatic medicine demonstrated the same theoretical eclecticism present in the Argentine group (Guedes, Rangel, Camargo Jr., 2020).

On the other hand, in a similar way to the theoretical production, the project of institutionalization of psychosomatic medicine in hospitals was developed, as occurred with the Psychosomatic Service at the Children’s Hospital in Argentina. In 1958, under the direction of Perestrello, a Center for Psychosomatic Medicine was founded in the Santa Casa de Misericordia linked to a university hospital of the Universidade do Brasil. In this way, teaching hospitals became a privileged space for the growth of the Brazilian psychosomatic movement, especially in Rio de Janeiro, with the work developed by psychoanalytic doctors (Guedes, Rangel, Camargo Jr., 2020, 2022).

Consequently, Perestrello and the psychoanalytic doctors of Rio de Janeiro maintained a double institutional link: with the teaching hospital and with the psychoanalytic society, traditionally the Brazilian Society of Psychoanalysis of Rio de Janeiro, recognized by IPA in 1959.

## Final considerations

The proposal that articulated psychoanalysis to medicine propagated by the Chicago Psychoanalytic Institute exerted a clear influence on the process of institutionalization of psychoanalysis in Argentina, in the 1940s and 1950s. Franz Alexander’s approaches to the emotional factors in somatic illnesses had a strong impact on Argentine psychoanalysts, especially shown in the written production of the members of APA. Also, the Argentine group further extended the theoretical possibilities of dialogue between psychoanalysis and medicine by conceiving all diseases as psychosomatic. This theoretical breadth was reflected in the expansion of psychoanalytic clinical practice in Argentina.

The return of both humanism and politics is an aspect to highlight in the institutionalization of APA. Psychosomatic medicine seems to have contributed to the return of humanism within Argentine psychoanalysis, while APA assumed a certain political position of resistance, aiming to maintain its freedom in a context of authoritarianism and censorship. In this sense, APA defended its autonomy and fought against threats to its liberal values. In this way, in the period studied, the institutionalization of Argentine psychoanalysis managed to reconcile the membership criteria of IPA – bureaucratization and political neutrality – with the political principles of the members of APA (Plotkin, 2003).

Regarding the relations between Argentina and Brazil, the article shows evidence of an intense exchange between psychoanalysts from both countries. Even a group of Brazilian doctors completed their psychoanalytic training at APA. The return of this group to Rio de Janeiro as members of APA allowed the construction of the local founding project of the Brazilian Society of Psychoanalysis of Rio de Janeiro. As part of a foundational narrative, there is the historical version of Marialzira Perestrello that produces an “origin myth,” according to which her group would represent the “pioneers” of Brazilian psychoanalysis.

The historical reconstruction presented in this article finds evidence that the formation of psychosomatic medicine, in Brazil and Argentina, had a prominent place in the institutionalization of psychoanalysis in both countries. However, while in Argentina the dissemination and theoretical production were carried out by a group of psychoanalysts, in Brazil they concentrated on the activities of Danilo Perestrello. This finding could indicate that in Argentina the configuration of psychosomatics included the participation of many psychoanalysts and, therefore, it would have been a more collective phenomenon than in Brazil. It is possible to assume that this difference between both countries is related to the interests of Argentines to strengthen and expand psychoanalysis, especially the private psychoanalytic clinic until the end of the 1950s, while Perestrello supported an agenda that went beyond the institutionalization of psychoanalysis and even psychosomatics. For Perestrello, medicine should be transformed through the inclusion of the theoretical and practical perspectives of psychoanalysis and psychosomatics. In an ambitious project, like Perestrello’s, psychosomatics represented a powerful strategy in a path of profound transformation of the traditional medicine of the time.

A significant similarity between the processes of institutionalization of psychoanalysis in Argentina and Brazil was the dominance of Melanie Klein’s theses in the groups and societies linked to IPA, consolidating, from 1960 on, as an orthodox current in both countries (Oliveira, 2017; Plotkin, 2003). However, between 1940 and 1950, psychosomatic medicine developed a unique trajectory that allowed it to escape the strict “surveillance” of Klein and her followers in Buenos Aires and, later, in Rio de Janeiro.

It is plausible to affirm that the current psychosomatic medicine, within the IPA institutions in both countries, achieved relative independence from the English school to the extent that it used its theoretical references. This relative independence is initially observed in the strong influence of the Chicago Psychoanalytic Institute, and during the 1950s through the contributions of the French school and Michel Balint, as well as local authors who presented their interpretations of the problems involved psychosomatic diseases. In our opinion, investigating to what extent psychosomatic medicine represented a difficulty for the exercise of strict Kleinian surveillance and what strategies were developed to control this threat to the institutional order in Argentina and Brazil could become an interesting question for future historical research.

## NOTES

1 In this event, in a photograph, APA groups from São Paulo and Rio de Janeiro were recorded with Marialzira and Danillo Perestrello and Walderedo Ismael de Oliveira (Marchon, 2012).
