# Supplementary material for: Historical reconstruction of Latin American psychosomatic medicine: the exchange between psychoanalysts from Argentina and Brazil, 1942-1959
Source: Hist Cienc Saude Manguinhos. 2024 Oct 11;31:e2024046. doi: 10.1590/S0104-59702024000100046en (PMC11472782; doi:10.1590/S0104-59702024000100046en)
Supplement: Translation

# Translation: Reconstrucción histórica de la medicina psicosomática latinoamericana: el intercambio entre psicoanalistas de Argentina y Brasil, 1942-1959

**DOI:** 10.1590/S0104-59702024000100046

**Authors:** Carla Ribeiro Guedes, Adriana Kaulino

**Affiliations:** i Profesora, Departamento de Saúde e Sociedade/Instituto de Saúde Coletiva/Universidade Federal Fluminense. Niterói – RJ – Brasil carlaguedes@id.uff.br; ii Profesora, Facultad de Psicología/Universidad Diego Portales. Santiago – Chile adriana.kaulino@udp.cl

**Keywords:** Medicina psicosomática, Institucionalización, Psicoanálisis, Historia

## Abstract

Ese artículo realiza una reconstrucción de los orígenes de la medicina psicosomática en Latinoamérica a través de la historia de la institucionalización del psicoanálisis en Argentina y Brasil. El estudio enfoca el intercambio entre psicoanalistas argentinos y brasileños durante el período correspondiente a los años 1942-1959. Los análisis indican similitudes y diferencias entre los dos proyectos de difusión y producción teórica de la medicina psicosomática en ambos países. Las conclusiones apuntan a que el grupo argentino de psicoanalistas tenía la intención de fortalecer el psicoanálisis mientras que, en Brasil, el principal difusor de la psicosomática, Danilo Perestrello, sostenía una transformación profunda de la medicina tradicional.

En 1902 se iniciaron las reuniones entre los primeros discípulos de Sigmund Freud en las famosas “tardes psicológicas de los miércoles”. Se trataba de un grupo formado, en su mayoría, por médicos e intelectuales descontentos con las problemáticas relacionadas con la subjetividad humana en la entonces Viena victoriana. En 1908, este grupo pasó a llamarse Sociedad Psicoanalítica, y, en 1910, se fundó la Asociación Psicoanalítica Internacional [International Psychoanalytical Association, IPA] (Rose, 1992).

A lo largo de los años, con el creciente reconocimiento social de Freud y del psicoanálisis, se fundaron diversas sociedades psicoanalíticas en todo el mundo. A partir de 1920 se inició un complejo proceso de institucionalización del psicoanálisis en la Policlínica Psicoanalítica de Berlín (transformada en instituto), bajo la dirección de Max Eitingon. Debido a la gran demanda de tratamiento psicoanalítico, se produjo un cambio significativo en la formación profesional de los nuevos analistas, la cual adquirió una agilidad que la hizo más impersonal y estandarizada. Puede decirse que el carácter contestatario y rebelde del psicoanálisis mundial de las dos primeras décadas fue modificado a mediados de la segunda por tendencias más conservadoras y reaccionarias (Vetö Honorato, 2013).

A su vez, las persecuciones provocadas por el nazismo y el estallido de la Segunda Guerra Mundial produjeron una dispersión geográfica, doctrinal e institucional, desplazando su centro de producción desde la cultura científica centroeuropea hacia la anglosajona. La mayoría de los psicoanalistas judíos emigraron a EEUU, donde fundaron instituciones psicoanalíticas fuertemente subordinadas a la IPA (Plotkin, 2009; Vetö Honorato, 2013).

La IPA, bajo la presidencia de Ernest Jones (también presidente de la Sociedad Psicoanalítica Británica), junto con la Asociación Psicoanalítica Americana [American Psychoanalytic Association] pasaron a controlar fuertemente el psicoanálisis con una orientación que lo consideraba una ciencia neutral, pura y apolítica con un enfoque exclusivamente terapéutico. La diáspora psicoanalítica deja atrás el carácter cultural, social, humanista e incluso subversivo del psicoanálisis, burocratizándose y cerrándose en una rígida jerarquía organizacional (Roazen, 1973; Vetö Honorato, 2013).

En la región latinoamericana, la Asociación Psicoanalítica Argentina (APA) fue reconocida formalmente por la IPA en 1942. Acompañando las transformaciones posguerra, en las que el psicoanálisis perdió su carácter humanista en favor de una vertiente fuertemente clínica-terapéutica, la APA durante sus primeros años estuvo en sintonía con las orientaciones teóricas de EEUU e Inglaterra. Esta tendencia se articuló con el rápido crecimiento de la medicina psicosomática, basada en el modelo estadounidense (Plotkin, 2003).

Los trastornos psicosomáticos empezaron a ser abordados por el psicoanálisis en la primera década del siglo XX con Sandor Ferenczi y George Groddeck. En los años 1930, en el Instituto Psicoanalítico de Chicago, surgió la propuesta de la medicina psicosomática. El médico y psicoanalista húngaro Franz Alexander y la médica estadounidense Helen Flanders Dunbar son sus principales representantes. Juntos fundaron, en 1939, la primera revista dedicada específicamente a la temática, el *Journal of Psychosomatic Medicine*. Ambos, a su manera, contribuyeron a los estudios de los orígenes de las patologías basados en formulaciones psicoanalíticas (Birman, 1980).

Los trabajos de Dunbar (1943) afirmaban que cada enfermedad se correspondía a un perfil psicológico, construido a partir de experiencias infantiles básicas. Mientras que, para Alexander, las enfermedades estaban vinculadas a conflictos inconscientes, es decir, a un conflicto específico. Alexander abordó básicamente siete patologías: asma bronquial, úlcera gástrica, artritis reumatoide, rectocolitis ulcerosa, dermatosis, tirotoxicosis e hipertensión esencial (Alexander, French, 1948).

La tradición de la medicina psicosomática ejerció una profunda influencia en los psicoanalistas argentinos en el proceso de constitución de la APA, lo que se puede evidenciar en las publicaciones de Franz Alexander en el número inaugural de la *Revista de Psicoanálisis* (Alexander, 1943) y en el primer libro producido por la biblioteca de la APA (Alexander, French, 1948). Se considera que, en sus inicios, el enfoque teórico asociado al modelo de profesionalización del psicoanálisis, en EEUU, tuvo una repercusión significativa en los miembros de la nueva asociación (Plotkin, 2003; Dagfal, 2018).

Esta cadena de influencias continúa con el impacto del desarrollo del psicoanálisis institucional argentino sobre los grupos psicoanalíticos en otros países latinoamericanos como Brasil. Muchos psicoanalistas brasileños de primera generación, afiliados a la IPA, hicieron su formación en Buenos Aires, entre ellos algunos médicos de Río de Janeiro, como Danilo Perestrello, considerado el precursor de la medicina psicosomática en Brasil (Guedes, Rangel, Camargo Jr., 2020).

A pesar de la importancia de la corriente de la medicina psicosomática en los orígenes institucionales del psicoanálisis en Argentina y Brasil, aún no existen estudios que aborden la configuración de la psicosomática en los dos países desde una perspectiva histórica e institucional. Hasta ahora, las referencias a la psicosomática suelen estar ligadas a la historia del psicoanálisis situando sus raíces en los centros anglosajones o europeos. Por lo tanto, mientras en las últimas décadas han proliferado estudios históricos de diversas índoles teórico-metodológicas acerca del psicoanálisis, son escasos los desarrollos de narrativas que reconstruyan las particularidades históricas de la psicosomática en América Latina.

Considerando la importancia de la medicina psicosomática y la escasez de estudios sobre su historia en la región, el artículo realiza una reconstrucción de sus orígenes a través de la historia de la institucionalización del psicoanálisis en Argentina y Brasil. La investigación focalizada en la institucionalización del psicoanálisis corresponde a una estrategia metodológica cuyo objetivo es identificar la particular apropiación de la psicosomática en ambos países. En términos teóricos, el artículo comparte los argumentos según los cuales el psicoanálisis sería un sistema transnacional de ideas y de creencias (Porras, Báguena, 2020; Plotkin, 2017) que se integra a los “saberes psi” (Rose, 1996). Sus orígenes son variados, múltiples y situados socioculturalmente en espacios diferentes de la formación de las sociedades psicoanalíticas oficiales. En consecuencia, este trabajo se focaliza en reconstruir sólo uno de los procesos relevantes en la configuración histórica de la psicosomática: su relación con la institucionalización y los mecanismos de legitimación del psicoanálisis en los contextos sociopolíticos de Argentina y Brasil.

El estudio abarca el período que se inicia con la fundación, en 1942, de la APA y que termina con la creación, en 1959, de la Sociedad Brasileña de Psicoanálisis de Río de Janeiro.

Las fuentes primarias de información fueron revistas, periódicos, conferencias, artículos y libros publicados por psicoanalistas que abordaron temáticas relativas a la medicina psicosomática en Argentina y Brasil durante este periodo. En particular, fueron analizadas las producciones discursivas relacionadas a la medicina psicosomática presentes en periódicos, libros y otras publicaciones de la *Revista de Psicoanálisis* de la APA, así como conferencias, textos y libros del psicoanalista brasileño formado por la asociación argentina, Danilo Perestrello, durante el periodo de 1942 a 1959.

Asimismo, se utilizaron fuentes secundarias para la reconstrucción de la institucionalización del psicoanálisis en ambos países. Con esta finalidad, fueron revisados manuscritos y libros sobre la historia de la institucionalización del psicoanálisis en América Latina y en los EEUU (Roazen, 1973; Bálan, 1991; Oliveira, 2000, 2017; Russo, 2002; Plotkin, 2003; Facchinetti, Ponte, 2003; Dagfal, 2009, 2018; Vetö Honorato, 2013; Ruperthuz, 2015; Facchinetti, Castro, 2015; Briolotti, 2016). Finalmente, fueron analizadas fuentes secundarias que permitieron comprender aspectos de los contextos sociales y políticos relevantes durante el periodo abordado por el estudio (Plotkin, 2009; Fiorucci, 2004).

## La institucionalización del psicoanálisis en Argentina

La medicina psicosomática fue uno de los temas que interesaron al joven pediatra Arnaldo Rascovsky, quien en los años 1930 promovió en su consultorio, en los domingos, una reunión informal para amigos y familiares, junto a su esposa Matilde Wenceblatt, con el fin de leer y debatir las obras de Freud. Entre los participantes se encontraba la pareja Enrique Pichón-Rivière y Arminda Aberastury (Plotkin, 2003).

Los debates para formar una asociación psicoanalítica afiliada a la IPA comenzaron en la década siguiente, en 1940, inicialmente con el proyecto de transformar el psicoanálisis en una especialidad médica. Esta propuesta fracasó, pero dos años después se creó la APA en un movimiento liderado por el español Ángel Garma. Aunque todos los fundadores tenían el título de médico, en sus inicios también se aceptaban miembros no médicos, como las esposas (Balán, 1991; Plotkin, 2003).

El grupo original estaba formado por médicos con ideas frescas sobre el psicoanálisis, pero muy diferentes entre ellos. Rascovsky era un joven pediatra judío respetado por la clase media judía de Buenos Aires y trabajaba en el Hospital de Niños. Pichon-Rivière nació en Suiza, emigró a Argentina cuando era niño con sus padres, se licenció en medicina y mantuvo relaciones con los círculos artísticos de la vanguardia porteña. Cuando comenzó a asistir a las reuniones de estudio sobre el psicoanálisis ya era un joven psiquiatra que trabajaba en el Hospicio de las Mercedes y estaba bien establecido en el sistema público de atención psiquiátrica. Ángel Garma emigró a Buenos Aires en 1938, el mismo año en que terminó su formación psicoanalítica, en Berlín, con Theodor Reik como su analista didacta.

Garma era miembro de la Asociación Internacional y llegó a Buenos Aires con importantes credenciales psicoanalíticas. Céles Cárcamo era un médico argentino, quien conoció a Garma mientras realizaba su formación en la Asociación Psicoanalítica de París. En 1939, Cárcamo regresó definitivamente a Buenos Aires con un título proporcionado por la IPA (Balán, 1991; Plotkin, 2003).

En 1942, la APA recibió el reconocimiento provisional de la IPA, mientras que su afiliación oficial solo ocurrió en 1949, durante el primer congreso psicoanalítico de la posguerra realizado en Zurich (Balán, 1991; Plotkin, 2003).

Al principio, la APA intentó establecer vínculos más estrechos con el circuito médico local. Sin embargo, los psicoanalistas argentinos terminaron por mantenerse alejados del *establishment* médico, con raras participaciones en conferencias organizadas fuera del circuito psicoanalítico local (Plotkin, 2003). Entre estas excepciones, se encuentra la participación en conferencias en Río de Janeiro y São Paulo, así como en un congreso médico ocurrido en Río de Janeiro (Marchon, 2012; Dagfal, 2018).

En julio de 1943, ocho meses después de su fundación, la APA comenzó a publicar la *Revista de Psicoanálisis*. Esta revista fue un importante instrumento de difusión, legitimación y validación de la APA. El pequeño grupo de psicoanalistas de la nueva asociación, que se parecía a una “sociedad secreta”, necesitaba obtener reconocimiento en los círculos especializados (Dagfal, 2018). Hasta finales de los años 1950, en pocas ocasiones los trabajos de sus psicoanalistas aparecieron en publicaciones externas a la revista de la APA (Plotkin, 2003). Una vez más, Río de Janeiro escapó a la regla, cuando la *Revista Evolução Psiquiátrica* publicó un artículo de Arnaldo Rascovsky y Ludovico Rosenthal (1947) sobre el movimiento psicoanalítico latinoamericano que también fue publicado en la *Revista de Psicoanálisis* de la APA.

Según Plotkin (2003), se puede comprender este aislamiento institucional relacionándolo con las condiciones de organización y formación de la APA, así como por el estatus profesional de los psicoanalistas que estaban afiliados a una institución privada sin vínculos con la estructura médica tradicional. Pichon-Rivière era el único del grupo que tenía conexiones consolidadas con el establecimiento psiquiátrico de Buenos Aires. Por otra parte, los psicoanalistas miembros de la asociación estaban ligados a una organización internacional y formaban parte de una red de relaciones de alcance mundial.

El aislamiento institucional de la APA, así como el éxito de su desarrollo, no pueden entenderse fuera del contexto político de la época. Las décadas de 1930 y 1940 estuvieron marcadas por una serie de transformaciones políticas en Argentina. El golpe de Estado de 1930 puso fin a un periodo de estabilidad institucional, y fue acompañado de acontecimientos internacionales como la Guerra Civil Española, el advenimiento del nazismo en Alemania, la radicalización del fascismo en Italia y la Segunda Guerra Mundial. A nivel nacional, el golpe militar de 1943 produjo un clima de polarización con la formación de dos grupos políticos y culturales: uno liberal progresista y otro nacionalista-católico. El gobierno de Perón en 1945 amplió esta radicalización política. Sus oponentes formaban una coalición heterogénea, desde socialistas y comunistas hasta liberales e incluso algunos conservadores. La política llegó a tener un fuerte matiz afectivo, en el que la gente se dividía entre ser peronista o antiperonista (Plotkin, 2003).

El régimen peronista consideraba que el arte de vanguardia, el existencialismo y otras corrientes de pensamiento, consideradas modernas, eran antinacionales, antipopulares y, por tanto, antiperonistas. En este contexto histórico, el psicoanálisis fue entendido como parte del sistema de resistencia cultural al peronismo. Los primeros miembros de la APA se identificaban con la tradición liberal que estaba amenazada. Garma había participado en el Centro Republicano Español, Pichón-Rivière tenía una formación socialista, y Marie Langer había pertenecido al partido comunista en Viena. Aunque los psicoanalistas no eran perseguidos explícitamente, siempre tenían policías en las reuniones de la APA (Plotkin, 2003).

Otra consecuencia del entorno político argentino fue la pérdida de prestigio y legitimidad de las instituciones académicas. El gobierno militar de 1930 eliminó por decreto la autonomía universitaria, y con el golpe de 1943 se pusieron al frente de las universidades figuras de la derecha nacionalista y católica que, entre otras medidas, despidieron a un gran número de profesores por razones puramente políticas. La llegada del gobierno de Perón, en 1945, profundizó aún más la intervención del Estado en las universidades. Este escenario contribuyó al desarrollo de una intensa vida intelectual fuera del ámbito universitario. La APA ganó legitimidad al conseguir mantener su autonomía científica y cultural (Plotkin, 2003).

Ahora bien, aún en este escenario adverso, en los primeros años los miembros de la APA buscaron ampliar su radio de influencia. Las estrategias de difusión incluían la distribución gratuita de la *Revista de Psicoanálisis* entre médicos y otros profesionales liberales, así como una agresiva difusión en el exterior (Dagfal, 2018). Para los objetivos de nuestro trabajo, es interesante observar que el primer número de la revista, publicado en 1943, se inaugura con un artículo del médico y psicoanalista Franz Alexander, uno de los pioneros de la medicina psicosomática en el Instituto Psicoanalítico de Chicago.

## La medicina psicosomática en la Asociación Psicoanalítica Argentina

La posición estratégica del texto de Alexander, titulado “Aspectos psicológicos de la medicina”, en el número inaugural de la *Revista de Psicoanálisis*, así como *Factores psicógenos en el asma bronquial*, el primer libro publicado por la Biblioteca de Psicoanálisis de la APA, también de este autor pero esta vez en colaboración con Thomas French, ambos de 1943, reflejan el impacto causado por la medicina psicosomática en los psicoanalistas argentinos.

Una de las particularidades de las primeras etapas de recepción del psicoanálisis en EEUU fue su integración al discurso médico y universitario, así como su gran profesionalización limitada a la práctica médica. De este modo, el psicoanálisis era convertido en una disciplina médica absorbida por el *establishment* psiquiátrico, en el cual era bastante popular que los jóvenes psiquiatras realizaran una formación psicoanalítica oficial (Vetö Honorato, 2013).

Si bien la APA se mantuvo desvinculada, hasta fines de la década de 1950, del medio médico argentino, la profesionalización del psicoanálisis fue proyectada, en sus inicios, por sus fundadores como algo estrictamente médico y relacionado con la medicina psicosomática. Incluso, en 1948, la APA comenzó a exigir el título de médico a quienes quisieran practicar el psicoanálisis (Balán, 1991; Plotkin, 2003).

De este modo, la APA se acercó cada vez más al psicoanálisis del modelo estadounidense. La propuesta defendida por Alexander era el advenimiento de una nueva medicina con base psicoanalítica que rescatara la visión del ser humano como un todo unitario, y no como una enfermedad. Es curioso observar cómo la perspectiva de integración cuerpo-mente de la psicosomática introduce de nuevo una retórica humanista en el discurso psicoanalítico que quiere penetrar en el mundo de la medicina.

En el artículo de apertura de la *Revista de Psicoanálisis*, Alexander (1943, p.63) afirma: “El paciente, considerado de nuevo como un ser humano, con sus preocupaciones, temores, esperanzas y desesperaciones, como un todo indivisible y no solo como un portador de órganos – de un hígado o estomago enfermo – se está convirtiendo en legítimo objeto del interés médico”.

Se observa en este pasaje la aparición de una “medicina psicológica científica” que dedicaba cada vez más atención al papel causal de los factores emocionales en la enfermedad.

Durante un período que se extiende desde 1942 hasta finales de los años 1950, el grupo inicial de la APA se interesó por la investigación psicosomática, ya sea en enfoques teóricos o clínicos. Las dos primeras décadas estuvieron dominadas por la difusión de eventos y publicaciones sobre el tema. Entre los principales hitos, se incluyen las realizaciones del curso sobre problemas psicosomáticos, dictado en el Hospital de los Niños en 1943 (Dagfal, 2018), del congreso sobre “cefaleas” en 1953 y el simposio sobre “obesidad y trastornos de la alimentación” en 1955, en el que la mayoría de sus miembros presentaron trabajos (Aizenberg, 1982). Asimismo, se destaca en este periodo la publicación de la obra *Patología psicosomática* de Arnaldo Rascovsky (1948), primer libro sobre el área compilado por un miembro de la institución.

Los psicoanalistas argentinos partieron de una declaración de principios constituida por la idea básica de que toda enfermedad era psicosomática (Aizenberg, 1982). En este aspecto, los miembros de la APA diferían de la perspectiva propuesta por Alexander de que algunas enfermedades eran psicosomáticas y otras no: “Este justificado intento didáctico de Alexander no excluye, para nuestro criterio, de ninguna manera el concepto sobre la participación psicogénica en todos los cuadros patológicos, así como en la comprensión del individuo considerado normal” (Rascovsky, 1948, p.11).

El gran crecimiento de la propuesta de la medicina psicosomática al interior del psicoanálisis argentino en estas primeras décadas se explica, en gran medida, porque esta perspectiva ofrecía un campo casi ilimitado de intervenciones clínicas en las que la medicina tradicional no había tenido éxito (Plotkin, 2003).

Como ilustración, Arnaldo Rascovsky y Luis Rascovsky (1945) realizaron una investigación desde un enfoque psicoanalítico, con 116 casos de epilepsia infantil a lo largo de cinco años en la consulta externa del servicio de neuropsiquiatría y endocrinología del Hospital de los Niños de Buenos Aires. El abordaje consistió en entrevistas periódicas a los cuidadores de los niños que presentaban crisis epilépticas, con el objetivo de recoger datos ambientales y psicológicos sobre la dinámica familiar. Los autores concluyeron que la situación más constante que precipitaba las crisis epilépticas era generada por una organización familiar de profunda constitución neurótica, y en particular debido a que esos niños compartían cama con uno o ambos padres (o sustitutos). Observaron que la simple separación del niño de la cama conducía a la desaparición de las crisis epilépticas o a una reducción de su número e intensidad. Desde el punto de vista de la hipótesis psicodinámica, afirmaron que la elaboración de los estímulos experimentados depende de la capacidad del “yo” para hacer frente a la estimulación recibida:

El acceso ocurre en situaciones, en que el yo se halla abrumado por cantidades estimulantes instintivas que no puede canalizar por los sistemas de organización oral, anal o genital y cuya magnitud es suficientemente intensa como para exigir y provocar un tipo de descarga somática anterior aún a la organización oral; lo que equivale a decir que el ataque epiléptico constituye un patrón de reacción anterior a la integración del yo postnatal (Rascovsky, Rascovsky, 1945, p.627).

Siguiendo con la tendencia de abordar estudios clínicos y/o debates teóricos, se encuentran entre los libros y artículos publicados por los psicoanalistas de la asociación y sus invitados, estudios que abarcan una amplia gama de enfermedades relacionadas con diferentes especialidades médicas: asma (Racker, 1948), coriza (Ferrari, 1944), enfermedades dermatológicas (Allendy, 1946), epilepsia (Rascovsky, Rascovsky, 1945; Pichon-Rivière, 1944; Cesio, 1952), cefalea y jaqueca (Cárcamo, 1945; Garma, 1958), manifestaciones ginecológicas y sexuales (Cárcamo, Langer, 1944; Langer, 1951), problemas endocrinos (Rascovsky, 1948), tos (Fenichel, 1944) y úlceras gástricas (Garma, 1945, 1954).

Es interesante observar que en estas obras no se puede identificar la afiliación a ninguna corriente de pensamiento específica de la medicina psicosomática. Por ejemplo, en el artículo de 1948 “Conceptos básicos en medicina psicosomática”, en el que Pichon-Rivière (1983) se apoya en nociones de Alexander, Kubel y Fenichel, cada uno con perspectivas distintas o incluso antagónicas sobre el tema (Aizenberg, 1982).

La pluralidad de autores y enfoques teóricos utilizados en los artículos apunta al hecho de que la influencia de la corriente de la medicina psicosomática estadounidense, especialmente en la primera década de la APA, no correspondió de manera determinante a una afiliación a la Escuela Psicoanalítica de Chicago. Desde el primer número de la *Revista de Psicoanálisis* de la APA, el protagonismo de Franz Alexander fue compartido con el de Melanie Klein, la psicoanalista austriaca domiciliada en Inglaterra y que lideraba una poderosa corriente de seguidores en la Sociedad Psicoanalítica Británica. Durante las décadas de 1950 y 1960, Klein expandió su dominio hacia países latinoamericanos como Argentina, Brasil y Chile (Dagfal, 2018; Oliveira, 2017; Vetö Honorato, 2013). Sin embargo, aunque se produjo una hegemonía clínica de Klein en Buenos Aires, puede decirse que la medicina psicosomática argentina se mantuvo caracterizada por su eclecticismo teórico (Aizenberg, 1982).

En 1955, el golpe de Estado que derrotó a Perón produjo una renovación política y sociocultural en Argentina, con la democratización y modernización de la universidad y la expansión de las actividades de los psicoanalistas en los servicios de salud pública. En este nuevo contexto político, a fines de la década de 1950, miembros de la APA comenzaron a trabajar en ámbitos institucionales antes impensados, como la Facultad de Medicina de la Universidad de Buenos Aires y en hospitales públicos (Dagfal, 2009).

En consonancia con la expansión de las actividades de los psicoanalistas en los servicios hospitalarios se desarrolló la interconsulta médico-psicológica que abordaba especialidades médicas como ginecología y obstetricia, endocrinología, psicoprofilaxis quirúrgica, dermatología, cardiología y pediatría (Aizenberg, 1982), y nuevas propuestas como la pediatría psicosomática (Briolotti, 2016).

Las décadas de 1940 y 1950 también se caracterizan por la expansión de la APA en América Latina, con la formación de psicoanalistas de diferentes países y un estrecho intercambio entre los miembros de la APA y los psicoanalistas de Río de Janeiro (Russo, 2002).

## El intercambio entre psicoanalistas de Argentina y Brasil

A diferencia de lo que ocurrió en Argentina, donde los médicos psicoanalistas se encerraron en un grupo autorreferencial al interior de la asociación psicoanalítica, en Río de Janeiro los grandes nombres que componían el *establishment* psiquiátrico ya propagaban el psicoanálisis en el servicio público, vinculado a proyectos pedagógicos e higiénicos (Russo, 2002).

Al asumir el poder en 1930, Getúlio Vargas colocó en marcha un programa de modernización social y cultural intelectualmente mucho más inclusivo que el de Perón en 1945. El régimen de Vargas creó un Estado fuerte y centralizado, con la intención de trabajar con los intelectuales, independientemente de su tendencia ideológica, en un amplio programa de reformas políticas y culturales. Este programa correspondía a la imagen construida por muchos intelectuales, la mayoría de ellos procedentes de las familias de la élite brasileña, que se consideraban los mentores de un pueblo inculto e incapaz. Así, la introducción del psicoanálisis en las instituciones educativas oficiales y en los hospitales fue más accesible para los médicos y educadores brasileños de lo que había sido para los profesionales argentinos (Fiorucci, 2004).

En este contexto de los años 1940, algunos jóvenes psiquiatras vinculados al Servicio Nacional de Enfermedades Mentales estaban descontentos con la orientación oficial de ese órgano y con la enseñanza de la Facultad de Medicina, que consideraban anticuada. Las críticas recayeron sobre Henrique Roxo (1877-1969), profesor de psiquiatría desde 1911 y responsable por introducir la teoría freudiana en sus cursos universitarios (Facchinetti, Ponte, 2003).

En 1944, este grupo de psiquiatras creó el Centro de Estudios Juliano Moreira (CEJM). Entre sus fundadores se encontraban Danilo Perestrello y Walderedo Ismael de Oliveira, a los que se unieron posteriormente Marialzira Perestrello y otros psiquiatras. El CEJM tenía como objetivo reunir a los interesados en el estudio de la obra de Freud e implementar la formación de psicoanalistas en Río de Janeiro, con base en las normas de la IPA. En este sentido, sus miembros trabajaban con dos alternativas: traer a un psicoanalista didacta a Río de Janeiro o bien realizar una formación analítica en el extranjero (Marchon, 2012).

Inicialmente, el grupo buscó atraer a un didacta de la APA que estuviera interesado en emigrar a Río de Janeiro. Arnaldo Rascovsky y Ángel Garma fueron invitados. Aunque la propuesta no obtuvo el resultado esperado, ambos psicoanalistas vinieron a Río de Janeiro para una serie de conferencias para el CEJM. Rascovsky en julio de 1945, y Garma en enero de 1946. Rascovsky también se encontró con el grupo de psicoanalistas que participaba del proceso de reconstitución de la sociedad de psicoanálisis de São Paulo (Marchon, 2012; Dagfal, 2018).

En su conferencia en Río de Janeiro, Arnaldo Rascovsky (1947) se refirió a la formación de la IPA en Europa, EEUU y Argentina. Mostró un gran entusiasmo por el modelo de formación estadounidense vinculado a las escuelas de medicina y a la profesión de médico, señalando la posibilidad de un camino similar en América Latina con el desarrollo de la medicina psicosomática en los sectores médicos. A su vez, Garma, en su propia conferencia y mostrando sintonía con su colega, también abordó temas de medicina psicosomática (Marchon, 2012).

La presencia de los analistas argentinos en el país reforzó los ánimos para buscar un analista didacta extranjero que quisiera venir a Río de Janeiro. Garma asesoró al grupo en su invitación a Georg Gerö. También se invitó a Marie Langer y al psicoanalista francés Daniel Lagache. Sin embargo, ninguna de las propuestas prosperó (Marchon, 2012).

El primer Congreso Interamericano de Medicina, celebrado en Río de Janeiro, en 1946, fue una nueva oportunidad para el intercambio entre psicoanalistas de Brasil y Argentina. Sobre los preparativos de este evento, Marie Langer (1964, p.89-90) comentó los esfuerzos de uno de los fundadores de la APA para difundir el psicoanálisis entre los brasileños: “Rascovsky, un hombre muy capaz para la propaganda y para convencer, sugirió que todos fuéramos a participar en el evento”.

De hecho, participaran del congreso varios psicoanalistas argentinos: Ángel Garma, Céles Cárcamo, Marie Langer, Matilde Wencelblat, Eduardo Krapf, Enrique Pichon-Rivière, Arnaldo y Luis Rascovsky, Alberto Taglieferro, Arminda Aberastury, Flora Scolni, Teodoro Schlossberg, Horacio Garcia Vega, además del mecenas de la APA, Francisco “Paco” Muñoz (Marchon, 2012; Dagfal, 2018). En el congreso, el grupo argentino presentó 17 trabajos contrastando con los tres expuestos por los brasileños Durval Marcondes, Virginia Bicudo y Darcy de Mendonça Uchôa, todos ellos supervisados por la analista didacta Adelheid Koch, del grupo de São Paulo, el cual había recientemente obtenido el reconocimiento provisorio de la IPA (Dagfal, 2018; Oliveira, 2017). Este movimiento de los argentinos parece demostrar la inversión política de la APA en la difusión del psicoanálisis en Brasil, especialmente en Río de Janeiro.^1^

En este mismo congreso, Danilo Perestrello (1987) realizó una conferencia sobre medicina psicosomática titulada “Importancia del factor psicológico en la etiopatogenia de las úlceras gastroduodenales”*.* En esta presentación, explicaba la génesis de las enfermedades somáticas a partir de conflictos inconscientes, utilizando nociones basadas en las teorías psicoanalíticas de Franz Alexander y del argentino Ángel Garma. De este modo, observamos en el contexto brasileño la formación de un marco teórico que establecía las bases de las enfermedades orgánicas a partir de la teoría psicoanalítica, combinando las contribuciones del Instituto Psicoanalítico de Chicago con las de los psicoanalistas de la APA. El éxito de la visita de analistas argentinos al CEJM el año anterior, con la difusión de las nociones de medicina psicosomática, empezaba a dar sus primeros frutos.

El fortalecimiento de las relaciones entre los grupos de Río de Janeiro y Argentina, junto con la dificultad de traer a un analista didacta extranjero, provocaron que algunos participantes del CEJM buscasen su traslado al exterior. Este fue el caso de Alcyon Baer Bahia, médico del Servicio Nacional de Enfermedades Mentales que había ido a Buenos Aires en 1945 a realizar su formación en la APA. Desde 1944 ya se encontraban en formación en la APA Mário Martins, psiquiatra de Porto Alegre, y su esposa, Zaira Martins.

Por su parte, Danilo Perestrello, Marialzira Perestrello y Walderedo Ismael de Oliveira aprovecharon el congreso de Río de Janeiro para acordar, con los didactas de la APA presentes en el evento, el inicio de sus análisis (Facchinetti, Ponte, 2003). En 1946, el matrimonio Marialzira y Danillo Perestrello embarcó hacia Buenos Aires para iniciar sus respectivos análisis, a los que se unió Walderedo Ismael de Oliveira unos meses después, en febrero de 1947 (Marchon, 2012).

La formación en una asociación psicoanalítica afiliada a la IPA tenía reglas muy estrictas, jerárquicas y elitistas, ya que era muy costosa en términos de tiempo y dinero. En los años 1940 y 1950, los requisitos impuestos para la obtención del título de psicoanalista exigían unas trescientas horas de análisis didáctico, la asistencia y aprobación de seminarios durante tres o cuatro años, el tratamiento de dos o tres casos completos bajo la supervisión de un analista didacta y la presentación de una monografía. Después de todo este proceso, el candidato podía ser aceptado como miembro asociado, y sólo tras unos años más de práctica y nuevos trabajos teóricos podía ser admitido finalmente como miembro de pleno derecho a la asociación. El pináculo de la jerarquía psicoanalítica consistía en ser elegido miembro didacta, es decir, en analista que estaba autorizado a formar a otros candidatos a psicoanalistas, sin tener que estar él mismo necesariamente en análisis (Plotkin, 2003).

Los cuatro jóvenes médicos de Río de Janeiro habían obtenido una licencia financiada por el Servicio Nacional de Enfermedades Mentales, lo que les permitió cubrir los altos costos de la formación analítica y dedicarse exclusivamente a sus calificaciones (Facchinetti, Ponte, 2003). Tras la formación, comenzaron los análisis didácticos y las supervisiones. Danilo Perestrello fue analizado por Cárcamo y supervisado por Pichon-Rivière y Marie Langer; Marialzira Perestrello tuvo a Pichon-Rivière como analista y fue supervisado por Garma y Cárcamo; Walderedo tuvo a Marie Langer como analista y a Garma y Arnaldo Rascovsky como supervisores; y Bahía fue analizada inicialmente por Garma (el análisis fue interrumpido) y posteriormente por Cárcamo, con la supervisión de Luis Rascovsky (Marchon, 2012).

A primera vista, llama la atención la repetición de los mismos nombres como analistas y supervisores. Esta situación también se dio en el Instituto APA, en el que los seminarios fueron impartidos por un selecto grupo formado por Arnaldo Rascovsky, Enrique Pichon-Rivière, Angel Garma, Céles Cárcamo, Mary Langer, Arminda Aberastury, Luis Rascovsky, Alberto Talliaferro, Luiza Alvarez de Toledo, Teodoro Schlossberg y E. Racker (desde 1949) (Marchon, 2012).

Dentro de cada asociación, los analistas tenían un enorme poder. Al mismo tiempo que administraban los análisis didácticos, eran supervisores, profesores y evaluadores de los candidatos. En una asociación pequeña, este tipo de organización podía generar situaciones confusas, ya que un analista didacta que conocía los secretos más íntimos de un candidato era quien evaluaba su rendimiento académico (Plotkin, 2003).

Este fue el escenario en el que los analistas de Río de Janeiro realizaron su formación en la APA. Además de los seminarios obligatorios centrados en temas relacionados con la teoría y la técnica psicoanalíticas, la aplicabilidad de la medicina psicosomática estaba presente en el Servicio de Psicosomática del Hospital de los Niños, donde Bahia era asistente (Marchon, 2012). Sin embargo, fue Danilo Perestrello quien se convirtió en el principal responsable de llevar el legado de las contribuciones psicoanalíticas a la medicina en Brasil (Guedes, Rangel, Camargo Jr., 2020).

Estando aún en formación en Buenos Aires, en 1948, Perestrello comenzó a difundir las nociones de la medicina psicosomática con una ponencia sobre las emociones y la colitis, en el ámbito estrictamente médico latinoamericano en un evento en Buenos Aires (ver Perestello, 1958a).

## La institucionalización del psicoanálisis en Brasil

Danilo y Marialzira Perestrello regresaron a Brasil en 1949, Alcyon B. Bahía y Walderedo Ismael de Oliveira en 1950, cuando crearon un grupo que pasó a ser conocido en los círculos psicoanalíticos como “los argentinos” (Marchon, 2012).

Por otra parte, recién en 1948 se había iniciado la formación de psicoanalistas en Río de Janeiro, primero con la llegada del psicoanalista Mark Burke, un judío polaco miembro de la Sociedad Psicoanalítica Británica, y, más tarde, con la de Werner Kemper, quien era miembro del Instituto de Berlín, incluso después de que este fuera “arianizado” por el Estado nazi. En 1936, tras varios actos de persecución contra los judíos que practicaban el psicoanálisis y contra la propia institución del psicoanálisis, el gobierno nazi obligó a la Sociedad Psicoanalítica Alemana a desafiliarse de la IPA y a unirse al Instituto Göring (Instituto Alemán de Investigación Psicológica y Psicoterapia). Los términos “psicoanálisis”, “psicoanalista” y todo el vocabulario psicoanalítico fueron eliminados de la escena pública. A pesar de que se les prohibió la práctica del psicoanálisis, tanto en la docencia como en la clínica, varios psicoanalistas siguieron en el Instituto Göring. Werner Kemper fue uno de ellos. Después de permanecer 12 años sometiéndose a las exigencias del régimen nazi, mediante el alegato de que así estaría protegiendo el psicoanálisis, Kemper llegó a Río de Janeiro con su esposa, Kattrin Kemper, para encargarse de la creación de una sociedad oficial de psicoanálisis (Russo, 2002).

Dos años después de la llegada de los dos analistas didactas, se produjo una primera disidencia. El grupo de Kemper rompió con el grupo vinculado a Burke, generando así dos sociedades de formación en una ciudad en la que aún no existía una asociación oficial. Las acusaciones entre los dos grupos eran mutuas y las razones de la ruptura se atribuyeron a cuestiones políticas relacionadas con el origen judío de Burke y con la conducta de Kemper durante el régimen nazi, así como a la disputa entre las escuelas alemana e inglesa de psicoanálisis. De todos modos, lo que sí se puede afirmar con certeza es que se inició una disputa entre los dos grupos por el reconocimiento oficial de la IPA (Russo, 2002).

El grupo de Kemper buscó apoyo en la Sociedad Brasileña de Psicoanálisis de São Paulo para el reconocimiento de su propio grupo de estudio. La Sociedad Brasileña de Psicoanálisis había sido fundada por Durval Marcondes, en 1927, en la capital paulista. Sin embargo, la sociedad se disolvió poco tiempo después. El proceso de su reconstitución se inició en 1937, con la llegada a São Paulo de la psicoanalista alemana Adelheid Koch, vinculada al Instituto Psicoanalítico de Berlín. De origen judía, Koch sufrió la persecución nazista y terminó aceptando la propuesta de Ernest Jones de emigrar para la capital paulista con la finalidad de organizar el grupo de psicoanálisis local y asumir la función de analista didacta. En el año subsiguiente, se iniciaron los primeros análisis didactas de América Latina. El reconocimiento provisorio como grupo de estudios por la IPA ocurrió en 1946. Su reconocimiento definitivo como sociedad psicoanalítica afiliada a la IPA acontecerá en 1951 (Oliveira, 2000; Facchinetti, Ponte, 2003).

El apoyo del grupo de Koch fue el primer paso en la constitución de una sociedad carioca de formación que obtuvo, en 1955, la autorización para convertirse en la Sociedad Psicoanalítica de Río de Janeiro. A su vez, las personas vinculadas a Burke (quien regresó a Inglaterra en 1953) se unieron a los analistas formados en Argentina y en 1957 fueron reconocidas por la IPA como un grupo de estudio. Dos años después, en 1959, este grupo fue reconocido como Sociedad Brasileña de Psicoanálisis de Río de Janeiro. A este último grupo pertenecían Marialzira y Danilo Perestrello (Russo, 2002).

El proyecto del matrimonio Perestrello incluyó la ocupación de la Sociedad Brasileña de Psicoanálisis de Río de Janeiro y la reivindicación de una posición de liderazgo en la historia del psicoanálisis en Río de Janeiro. En un libro escrito por Marialzira Perestrello (1987), aparece una narrativa en la que el movimiento psicoanalítico se divide entre los “predecesores” y los “pioneros”. El primer grupo estaría formado por los difusores del psicoanálisis en Río de Janeiro y el segundo compuesto por los responsables de introducir la formación psicoanalítica en Brasil a partir de las orientaciones de la IPA. Esta diferenciación pretendía demostrar que los “pioneros” habían sido más importantes en la definición de la teoría, la práctica y el estudio del psicoanálisis en Río de Janeiro que sus predecesores, o sea, los psiquiatras psicoanalíticos que, en la década de 1920, fueron los responsables por las primeras traducciones de la obra de Freud en el país. Al construir esta narrativa histórica, los Perestrello y su grupo se colocaron en la posición de los pioneros comprometidos con la difusión del “verdadero” psicoanálisis (Facchinetti, Castro, 2015).

Es interesante observar cómo esta narrativa genera un “mito de origen” (Danziger, 1979) para el psicoanálisis brasileño. El término mito indica la debilidad de una interpretación histórica que pretende ser verdadera y legitimar, a través del pasado, alguna situación del presente. Los mitos de origen son reiterados en las interpretaciones históricas tradicionales del psicoanálisis. Desde esta perspectiva historiográfica, se atribuye al psicoanálisis una “vida organizacional legítimamente reconocida” (Ruperthuz, 2021, p.718). Asimismo, en las historiografías tradicionales es frecuente el uso del concepto de pionero, en particular, en las historias locales del psicoanálisis. La apelación a los pioneros contribuye a demarcar fronteras entre la prehistoria y el inicio de la historia del psicoanálisis oficial. Los pioneros se distinguen tanto de sus antecesores como de los disidentes por su fidelidad y contribución teórica a un supuesto verdadero psicoanálisis freudiano. En consecuencia, estas narrativas históricas suelen resaltar la importancia de los pioneros en la fundación de las sociedades psicoanalíticas oficialmente reconocidas por la IPA.

La historia elaborada por Marialzira Perestrello es un claro ejemplo de esta estrategia historiográfica de legitimación del psicoanálisis oficial. Sin embargo, en este caso el mito de origen ha sido develado por diversos trabajos que entregan evidencias de que las diferencias entre “pioneros*”* y “predecesores” son altamente cuestionables (Facchinetti, Castro, 2015; Castro, 2017). Así, la historia que escribe Marialzira Perestrello sería una estrategia en la lucha por establecer los verdaderos herederos o representantes de una propuesta teórica en la cual la autora atribuye a su propio grupo el protagonismo del psicoanálisis en Río de Janeiro (Russo, 1993).

## La medicina psicosomática en Río de Janeiro

Mientras los diferentes grupos se esforzaban por obtener el reconocimiento institucional de la IPA para la fundación de la sociedad de psicoanálisis, Danilo Perestrello se convertía en el principal difusor de la medicina psicosomática brasileña, asumiendo el protagonismo y prácticamente la exclusividad, en los años 1940 y 1950, de las publicaciones sobre el tema (Guedes, Rangel, Camargo Jr., 2020). Esta es una diferencia con respecto al grupo argentino, pues la mayoría de los psicoanalistas de la APA escribieron sobre psicosomática en su plataforma oficial de difusión, la *Revista de Psicoanálisis* (Dagfal, 2018).

En el caso de Perestrello, sus estrategias para visibilizar la propuesta de la medicina psicosomática fueron sus conferencias en eventos, congresos nacionales e internacionales del área médica, además de clases y comunicaciones orales en universidades y hospitales, las cuales fueron compiladas en el libro *Medicina psicossomática*, publicado en 1958, considerado el trabajo inaugural de este campo en el contexto brasileño (Guedes, Rangel, Camargo Jr., 2020).

A lo largo de estas dos décadas, Perestrello se dedicó a realizar aportaciones psicoanalíticas para explicar la causalidad de diferentes patologías somáticas (Perestrello, 1958c), como las úlceras (Perestrello, D., 1987), las cefaleas (Perestrello, 1958d, 1954), las dolencias dermatológicas (Perestrello, 1958e) y las enfermedades digestivas (Perestrello, 1958f, 1958g).

En estos trabajos se utilizaron como referencias teóricas los maestros argentinos Garma, Rascovsky, Pichon-Rivière y Cárcamo, así como los estadounidenses Alexander y Dunbar, aunque también se nota en su obra de los años 1950 (Perestrello, 1958d) la influencia del psicoanalista francés Pierre Marty, que, junto a Michel de M’Uzan, creó la Escuela de Psicosomática de París. La escuela francesa rompe con la escuela psicoanalítica de Chicago al concebir el hecho psicosomático desvinculado de la nosografía médica y de la investigación de las personalidades correspondientes a las enfermedades psicosomáticas (Marty, M’Uzan, 1963).

Más allá de las cuestiones clínicas, Perestrello defiende el proyecto de que el psicoanálisis puede contribuir al surgimiento de una “nueva medicina”. Según él, la medicina y la práctica clínica debían superarse mediante una transformación gradual del modelo médico organicista hacia una medicina psicosomática (Guedes, Rangel, Camargo Jr., 2020). En este nuevo paradigma, el objeto de la medicina pasaría a ser el “paciente como persona”, y la vida psíquica del paciente se convertiría en un valor para entender la enfermedad somática (Perestrello, 1958b). De este modo, la superación de la medicina organicista se haría por una vía humanista a través de una medicina de base psicoanalítica (Perestrello, 1958b).

Así, se puede observar que la propuesta de Franz Alexander de retomar la perspectiva humanista del psicoanálisis para dialogar con la medicina es un argumento central en el proyecto de Perestrello.

En 1956, en su primer curso de medicina psicosomática en una cátedra de psiquiatría de la Facultad de Medicina de la Universidade do Brasil (actual Universidade Federal do Rio de Janeiro), Perestrello presentó el modelo estadounidense de inclusión del psicoanálisis en las facultades de medicina. El psicoanalista argumentaba que, para modificar la práctica médica, era necesario invertir en la enseñanza e institucionalización de la medicina psicosomática como lo hicieron los EEUU: “En la Universidad de Columbia, por ejemplo, se creó la ‘Clínica de Medicina Psicosomática y Psicoanálisis’ con fines de enseñanza e investigación, bajo la dirección de profesores del Instituto Psicoanalítico de Nueva York; y hoy en día, la mayoría de las facultades de medicina norteamericanas enseñan psiquiatría en todos los años del curso de medicina” (Perestrello, 1958h, p.32; énfasis en el original).

Así, Perestrello discute la idea de crear la disciplina de psicosomática o psicología médica en los cursos de medicina, con el fin de dotar al estudiante de conocimientos psicológicos básicos. Sin embargo, para él esta medida sería solo una transición, porque en el futuro la medicina sería psicosomática y todos los cursos de medicina seguirían esta orientación.

Perestrello también se dedicó a sensibilizar a los médicos para que la propuesta de la medicina psicosomática pudiera aplicarse en la medicina clínica. El autor expuso seis principios de la medicina psicosomática que deberían guiar la práctica clínica: (1) el objeto de estudio del médico es el hombre enfermo y no la enfermedad; (2) no hay enfermedades locales, toda enfermedad es general y afecta al individuo en su conjunto; (3) el individuo aislado es una abstracción y solo puede concebirse en su entorno; (4) los estados emocionales pueden perturbar el funcionamiento de cualquier órgano y son tan eficaces como los estímulos físicos en la producción de modificaciones somáticas; (5) los conflictos inconscientes son los principales responsables de los síntomas somáticos; (6) los trastornos emocionales pueden provocar lesiones orgánicas (Perestrello, 1958b).

A lo largo de los años, el enfoque de la práctica médica adquirió nuevos contornos en la trayectoria de Perestrello y se relacionó también con una perspectiva humanista de la relación médico/paciente, especialmente a partir de la influencia del libro de Michael Balint (1958) *El médico, su paciente y la enfermedad.*

Si bien es cierto que, principalmente a partir de los años 1960, los referenciales teóricos y clínicos de Melaine Klein, junto a la lectura de la obra de Wilfred Bion, orientaron las prácticas de las sociedades brasileras vinculadas a la IPA (Oliveira, 2017), es interesante notar que los escritos de Perestrello sobre la medicina psicosomática demostraron el mismo eclecticismo teórico presente en el grupo argentino (Guedes, Rangel, Camargo Jr., 2020).

Por otra parte, de manera similar a la producción teórica, se desarrollaba el proyecto de institucionalización de la medicina psicosomática en los hospitales, como ocurrió con el Servicio Psicosomático en el Hospital de los Niños en Argentina. En 1958, bajo la dirección de Perestrello, se fundó un Centro de Medicina Psicosomática en la Santa Casa de Misericordia vinculado a un hospital universitario de la Universidade do Brasil. De esta forma, los hospitales de enseñanza se convirtieron en un espacio privilegiado para el crecimiento del movimiento psicosomático brasileño, especialmente en Río de Janeiro, con el trabajo desarrollado por los médicos psicoanalistas (Guedes, Rangel, Camargo Jr., 2020, 2022).

En consecuencia, Perestrello y los médicos psicoanalistas de Río de Janeiro mantuvieron un doble vínculo institucional: con el hospital escuela y con la sociedad psicoanalítica, tradicionalmente la Sociedad Brasileña de Psicoanálisis de Río de Janeiro, reconocida por la IPA en 1959.

## Consideraciones finales

La propuesta que articulaba el psicoanálisis a la medicina propagada por el Instituto Psicoanalítico de Chicago ejerció una clara influencia en el proceso de institucionalización del psicoanálisis en Argentina, en las décadas de 1940 y 1950. Los planteamientos de Franz Alexander sobre los factores emocionales presentes en las enfermedades somáticas tuvieron una fuerte repercusión entre los psicoanalistas argentinos, demostrada sobre todo en la producción escrita de los miembros de la APA. Asimismo, se puede afirmar que el grupo argentino extendió aún más las posibilidades teóricas de interlocución entre el psicoanálisis y la medicina al concebir todas las enfermedades como psicosomáticas. Esta amplitud teórica se reflejó en la expansión de la práctica clínica psicoanalítica en Argentina.

Un aspecto a destacar en la institucionalización de la APA es el retorno tanto del humanismo como de lo político. La medicina psicosomática parece haber contribuido al regreso del humanismo al interior del psicoanálisis argentino, en el mismo tiempo que la APA asumía una cierta posición política de resistencia, con el objetivo de mantener su libertad en un contexto de autoritarismo y censura. En este sentido, la APA defendió su autonomía y luchó contra las amenazas a sus valores liberales. De este modo, puede afirmarse que, en el periodo estudiado, la institucionalización del psicoanálisis argentino logró conciliar los criterios de afiliación de la IPA – burocratización y neutralidad política – con los principios políticos de los miembros de la APA (Plotkin, 2003).

Con respecto a las relaciones entre Argentina y Brasil, el artículo muestra evidencias de un intenso intercambio entre psicoanalistas de ambos países. Incluso un grupo de médicos brasileros realizó su formación psicoanalítica en la APA. El regreso de este grupo a Río de Janeiro como miembros de la APA permitió la construcción del proyecto local de fundación de la Sociedad Brasileña de Psicoanálisis de Río de Janeiro. Como parte de una narrativa fundacional, se encuentra la versión histórica de Marialzira Perestrello que produce un “mito de origen”, según el cual su propio grupo representaría a los “pioneros” del psicoanálisis brasileño.

La reconstrucción histórica expuesta en este artículo encuentra evidencias de que la conformación de la medicina psicosomática, en Brasil y Argentina, tuvo un lugar destacado en la institucionalización del psicoanálisis en ambos países. Sin embargo, mientras en Argentina la difusión y producción teórica fueron realizadas por un grupo de psicoanalistas, en Brasil se concentraron en las actividades de Danilo Perestrello. Este hallazgo podría indicar que en Argentina la configuración de la psicosomática incluyó la participación de muchos psicoanalistas y, por lo tanto, habría sido un fenómeno más colectivo que el ocurrido en Brasil. Asimismo, es posible suponer que esta diferencia entre ambos países se relaciona con los intereses de los argentinos por fortalecer y expandir el psicoanálisis, sobre todo la clínica psicoanalítica privada hasta fines de los años 1950, mientras que Perestrello sostenía una agenda que iba más allá de la institucionalización del psicoanálisis e incluso de la psicosomática. Para Perestrello, la propia medicina debería transformarse a través de la inclusión de las perspectivas teóricas y prácticas del psicoanálisis y de la psicosomática. En un proyecto ambicioso, como el de Perestrello, la psicosomática representaba una estrategia poderosa en un camino de transformación profunda de la medicina tradicional de la época.

Una significativa similitud entre los procesos de institucionalización del psicoanálisis en Argentina y Brasil fue el dominio de las tesis de Melanie Klein en los grupos y sociedades vinculadas a la IPA, consolidándose, a partir de 1960, como una corriente ortodoxa en ambos países (Oliveira, 2017; Plotkin, 2003). Sin embargo, entre 1940 y 1950, la medicina psicosomática desarrolló una trayectoria singular que le permitió escapar de la estricta “vigilancia” de Klein y sus seguidores en Buenos Aires y, posteriormente, en Río de Janeiro.

Es plausible afirmar que la corriente de la medicina psicosomática, al interior de las instituciones ipeístas en ambos países, logró una relativa independencia de la escuela inglesa, en la medida que utilizó referenciales teóricos propios. Esta relativa independencia se observa inicialmente en la fuerte influencia del Instituto Psicoanalítico de Chicago, y durante los años 1950 a través de las contribuciones de la escuela francesa y de Michel Balint, además de autores locales que presentaron sus propias interpretaciones acerca de las problemáticas que involucraban las enfermedades psicosomáticas. A nuestro juicio, el indagar en qué medida la medicina psicosomática representó una dificultad para el ejercicio de la estricta vigilancia kleiniana y qué estrategias se desarrollaron para controlar esta amenaza al orden institucional en Argentina y Brasil podría convertirse en una interesante pregunta para futuras investigaciones históricas.

## NOTAS

1 En este evento, en una fotografía, se registraron los grupos de la APA, de São Paulo y de Río de Janeiro con Marialzira y Danillo Perestrello y Walderedo Ismael de Oliveira (Marchon, 2012).
